# Current Knowledge and Adoption of Mobile Health Apps Among Australian General Practitioners: Survey Study

**DOI:** 10.2196/13199

**Published:** 2019-06-03

**Authors:** Oyungerel Byambasuren, Elaine Beller, Paul Glasziou

**Affiliations:** 1 Centre for Research in Evidence-Based Practice Bond University Robina, QLD Australia

**Keywords:** mobile health, smartphone, mobile apps, mHealth, smartphone apps, general practice

## Abstract

**Background:**

Mobile health (mHealth) apps can be prescribed as an effective self-management tool for patients. However, it is challenging for doctors to navigate 350,000 mHealth apps to find the right ones to recommend. Although medical professionals from many countries are using mHealth apps to varying degrees, current mHealth app use by Australian general practitioners (GPs) and the barriers and facilitators they encounter when integrating mHealth apps in their clinical practice have not been reported comprehensively.

**Objective:**

The objectives of this study were to (1) evaluate current knowledge and use of mHealth apps by GPs in Australia, (2) determine the barriers and facilitators to their use of mHealth apps in consultations, and (3) explore potential solutions to the barriers.

**Methods:**

We helped the Royal Australian College of General Practitioners (RACGP) to expand the mHealth section of their annual technology survey for 2017 based on the findings of our semistructured interviews with GPs to further explore barriers to using mHealth apps in clinical practice. The survey was distributed to the RACGP members nationwide between October 26 and December 3, 2017 using Qualtrics Web-based survey tool.

**Results:**

A total of 1014 RACGP members responded (response rate 4.6% [1014/21,884], completion rate 61.2% [621/1014]). The median years practiced was 20.7 years. Two-thirds of the GPs used apps professionally in the forms of medical calculators and point-of-care references. A little over half of the GPs recommended apps for patients either daily (12.9%, 80/621), weekly (25.9%, 161/621), or monthly (13.4%, 83/621). Mindfulness and mental health apps were recommended most often (32.5%, 337/1036), followed by diet and nutrition (13.9%, 144/1036), exercise and fitness (12.7%, 132/1036), and women’s health (10%, 104/1036) related apps. Knowledge and usage of evidence-based apps from the Handbook of Non-Drug Interventions were low. The prevailing barriers to app prescription were the lack of knowledge of effective apps (59.9%, 372/621) and the lack of trustworthy source to access them (15.5%, 96/621). GPs expressed their need for a list of safe and effective apps from a trustworthy source, such as the RACGP, to overcome these barriers. They reported a preference for online video training material or webinar to learn more about mHealth apps.

**Conclusions:**

Most GPs are using apps professionally but recommending apps to patients sparingly. The main barriers to app prescription were the lack of knowledge of effective apps and the lack of trustworthy source to access them. A curated compilation of effective mHealth apps or an app library specifically aimed at GPs and health professionals would help solve both barriers.

## Introduction

Over the past decade, smartphones have become an inseparable part of modern living, and mobile health (mHealth) apps have started to establish their place in health care [1]. If proven to help achieve measurable clinical improvements in patients’ conditions, mHealth apps can be officially prescribed or recommended by general practitioners (GPs) [2]. However, with 350,000 apps available in the medical and health and fitness categories in the major app stores, it is challenging for GPs to find prescription quality mHealth apps from the app stores themselves to use in their clinical practice [3]. To overcome this issue, a number of initiatives such as the National Health Service (NHS) Apps Library in the United Kingdom [4] and Health Navigator app library in New Zealand [5] have been set up to help doctors. In Australia, official effort to support app prescription does not exist yet, but there are small initiatives such as the Victorian Health Promotion Foundation’s Healthy Living Apps Guide aimed at the general public [6] and the Handbook of Non-Drug Interventions (HANDI) project by the Royal Australian College of General Practitioners (RACGP) [7], which is a repository of evidence-based nonpharmaceutical interventions for GPs.

Health care professionals’ use of mHealth apps and mobile technologies have been explored in several recent reports from the United States [8], United Kingdom [9], France [10], and Turkey [11]. At least half of the surveyed GPs, specialists, dieticians, and pharmacists reported to recommend mHealth apps to patients, except for the French study of GPs, which reported half that rate. Barriers perceived by the health professionals regarding mHealth integration to their clinical practice include a variety of issues from infrastructure related problems such as Wi-Fi coverage and interoperability with the existing medical software to more specific data security, content reliability, and a universal lack of awareness of the effective apps to use. All of this echoes the findings of an earlier systematic review by Gagnon et al [12], which summarized the barriers and facilitators to mHealth adoption by health care professionals from around the globe.

Australian GPs’ mHealth adoption has been explored only briefly as part of the annual technology survey by the RACGP since 2015. The purpose of this survey is to explore technological innovation and adoption in general practice, including mobile technology [13]. Following our qualitative study with GPs on the barriers and facilitators of mHealth app use in general practice, we collaborated with the RACGP to expand the mHealth section for 2017 to better understand the specific barriers to health app use in the wider Australian context. Thus, the objectives for this study were to (1) explore the knowledge and use of health apps of practicing GPs in Australia in more detail, (2) determine the barriers and facilitators to prescribing health apps in GP consultations in a wider cohort of GPs, and (3) explore the potential solutions to some of the barriers.

## Methods

We used the Checklist for Reporting Results of Internet E-Surveys, recommended by the Journal of Medical Internet Research as a reporting guide for this study [14]. The data for this study were collected as part of the 2017 RACGP Technology Survey, which was conducted using a Web-based survey tool Qualtrics (Qualtrics, Provo, UT) between October 26 and December 3, 2017 in Australia [15]. We used convenience sampling and the survey link was emailed to all RACGP members, which included GP trainees, fellows, and vocationally registered GPs, as well as practice managers and clinic owners. Only the GPs currently practicing in Australia were able to advance and answer all questions. For GP registrars and GPs who were not practicing currently or not practicing in Australia, the survey ended after the relevant questions. Ethics approval was obtained from the RACGP National Research and Evaluation Ethics Committee.

The previous year’s survey contained 6 questions regarding GPs’ use of mobile devices and mHealth apps out of 46 questions [13]. On the basis of the findings of our semistructured interview study with 20 GPs who explored the barriers and facilitators to using mHealth apps in practice, we collaborated with the RACGP to expand the mHealth section questions for the 2017 survey making them more specific and informative. The questions were pilot-tested with the coauthors and academic GP colleagues and refined iteratively. This paper reports the analysis of 16 questions pertaining to demographic information, mobile device, and health apps usage out of the total 50 questions (Textbox 1).

Data were extracted from Qualtrics and descriptive statistics were conducted using Microsoft Excel (2016). Answers to the open-ended questions were coded according to their common themes (OB), checked by a second author (EB), and then summarized. Participation in the survey was voluntary and participants who completed the survey were invited to enter a draw to win one of 2 gift cards worth Aus $50.

**Survey questions analyzed as part of this study.**

**Screening and demographics questions**

1. I am... (a general practitioner, a general practitioner registrar, other)

2. What is your role in the practice?

3. Where did you complete your training? (Australia, Overseas)

4. Do you currently practice in Australia? (Yes/No)

5. For how many years have you worked as a general practitioner? (1-5 years, 5-10 years, 10-20 years, 20-30 years, more than 30 years)

6. Please enter the postcode of your current practice location in which you spend the most time.

7. What is your age group? (Less than 35 years, 35-44 years, 45-54 years, 55-64 years, over 65 years)

**Mobile health section**

8. Do you use mobile devices in your day-to-day practice for patient-related work? (Yes/No)

9. I don't use mobile devices for patient-related work because... (choose all that apply)

- I am not confident in how to safely use mobile technology in my day-to-day practice
- I don't see how mobile technology can benefit my day-to-day practice
- My practice does not allow me to use my own mobile devices
- Other (please comment)

10. Which health apps do you use for yourself?

11. How often do you recommend the use of health apps to patients? (Daily, Weekly, Monthly, Rarely, Never)

12. Please share with us which health apps you have recommended.

13. Do you ever prescribe any of the following health apps to your patients (choose all that apply): (Quit Now: My QuitBuddy, Quit for You–Quit for Two, Sleepio, CBT-i Coach, SHUTi, Ankle, I do not prescribe the apps above)

14. Please rate the following barriers for health app integration into your daily clinical practice (where 1 is the most important barrier and 7 is the least important):

- lack of knowledge of effective apps
- lack of a trustworthy source to access effective apps
- lack of patient digital literacy
- lack of access to mobile devices
- lack of patient interest
- lack of practice incentives
- lack of understanding about benefits
- others (please specify)

15. What would help you to recommend health apps to patients more often?

16. How would you like to receive training on the use of effective health apps, including app evaluation? (eg, webinars, animations, podcasts)

## Results

Of the 39,380 people on the RACGP mailing list, who were emailed the survey link, 21,884 were currently practicing GPs and 1014 of them responded to the survey (4.6%). The survey completion rate was 61.2% (621/1014). The median age was 51.4 years and the median years practiced was 20.7 years. Age and geographical distribution by state were representative of Australian GP workforce statistics [16]. About a quarter of the survey responders were trained overseas, which was half the rate of the national statistics (Table 1).

A half of the GPs reported not using mobile devices for patient related work (n=312; 50%). The main reasons for this included GPs not seeing how mobile technology could benefit their day-to-day practice (n=136; 39%), not being confident on how to use mobile technology safely in daily practice (n=68; 20%), and the practice they worked for did not allow the use of personal mobile devices in practice (n=51; 15%). Other reasons (n=91; 26%) included not wanting to use personal mobile devices in consultations, their desktop computer being sufficient and more convenient, and not needing to use mobile devices altogether. About two-thirds of the GPs used health apps themselves (n=440; 64%), mostly in the form of point-of-care references such as UpToDate, eTG, Medscape (n=298; 25%), and medical calculators (n=137; 11%).

**Table 1:** Participants’ demographics.

Groups		This study, n (%)	National data (n=25,825), n (%)
**Age (years; n=621)**			
	<35	46 (7.4)	2376 (9.2)
	35-44	126 (20.3)	6361 (24.6)
	45-54	196 (31.6)	7327 (28.4)
	55-64	174 (28.0)	6637 (25.7)
	65+	79 (12.7)	3124 (12.1)
**Practice (years; n=621)**			
	<5	82 (13)	—^a^
	5-10	81 (13)	—
	10-20	136 (22)	—
	20-30	166 (27)	—
	>30	156 (25)	—
**General physician training (n=621)**			
	Overseas	144 (23.2)	13207 (51.1)
	Australia	477 (76.8)	12621 (48.9)
**Geographic distribution (n=844)**			
	New South Wales	235 (27.8)	8468 (32.8)
	Victoria	215 (25.5)	6506 (25.2)
	Queensland	174 (20.6)	5525 (21.4)
	Western Australia	78 (9.2)	2411 (9.3)
	South Australia	70 (8.3)	1873 (7.3)
	Tasmania	37 (4.4)	510 (2.0)
	Northern Territory	21 (2.5)	212 (0.8)
	Australian Capital Territory	14 (1.7)	320 (1.2)

^a^Not applicable.

**Figure 1:**
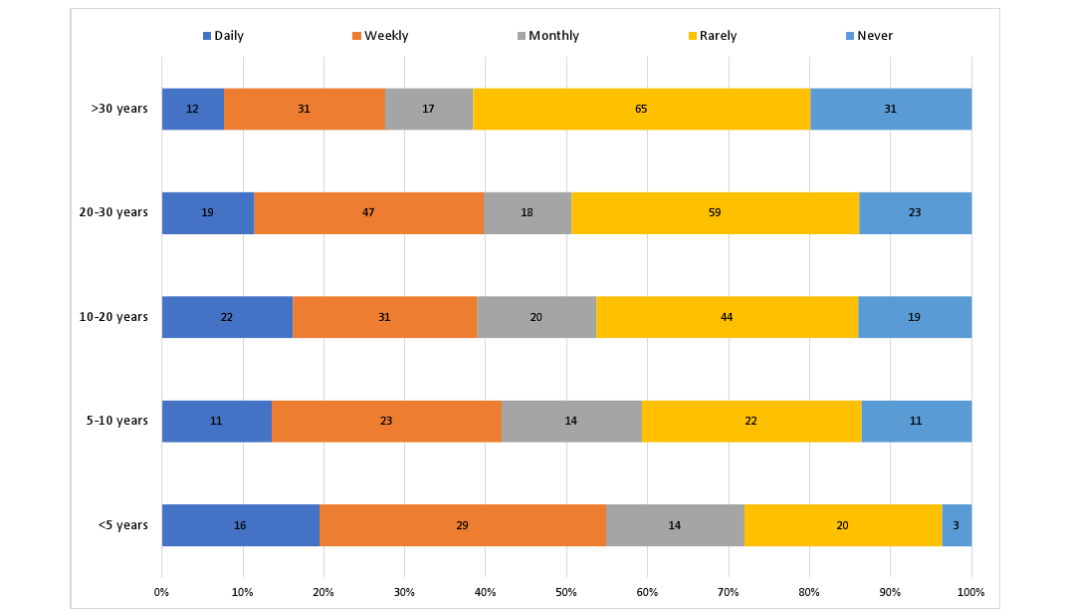
Frequency of app recommendation by years practiced.

A little over half of the GPs recommended apps for patients daily (n=80; 13%), weekly (n=161; 26%), or monthly (n=83; 13%). The other half rarely (n=210; 34%) or never (n=87; 14%) recommended apps. Figure 1 shows that the app recommendation frequency appears to decrease with the number of years practiced as a GP. GPs most commonly recommended mindfulness and mental health (n=337; 33%), diet and nutrition (n=144; 14%), exercise and fitness (n=132; 13%), and women’s health (n=104; 10%) related apps to patients. Examples of the specific apps they use included Smiling Mind, Headspace, MyFitnessPal, and Easy Diet Diary.

The question about evidence-based apps from the HANDI project revealed that smoking cessation apps were reported as prescribed 119 times, insomnia apps 39 times, and the ankle exercise app 7 times. However, majority of the GPs did not recommend any of the 6 apps that are currently offered in HANDI (n=417; 72%).

**Figure 2:**
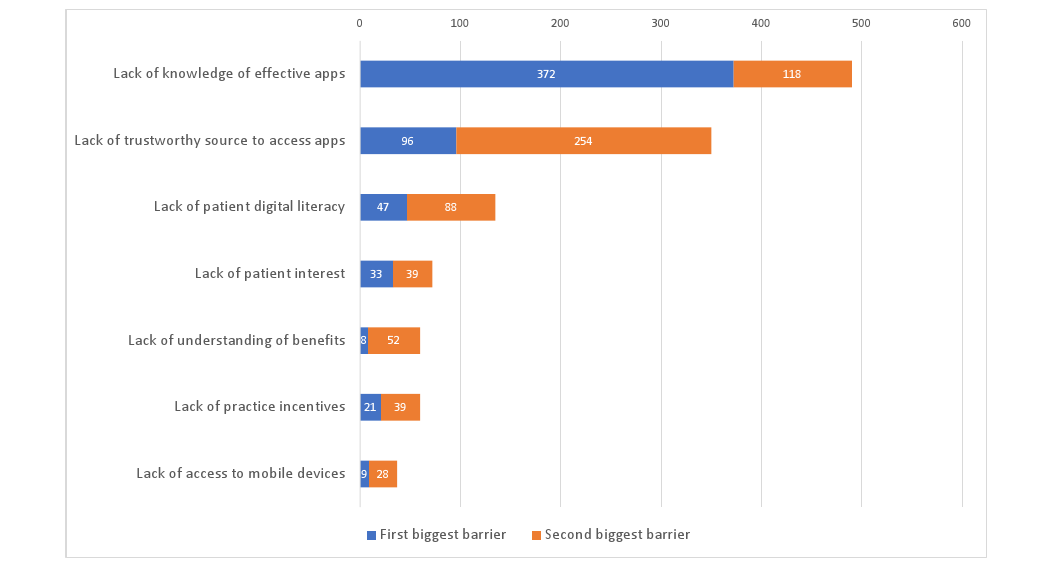
Barriers to app prescription. Numbers inside the bars show how many times the option was rated as the first and the second biggest barrier.

GPs also rated the barriers to integrating health apps into their daily practice. The prevailing barriers were the lack of knowledge of effective apps (n=372; 60%) and the lack of trustworthy source to access them (n=96; 15%). Figure 2 shows the ranking of barriers rated by the combination of the first and second most important barriers. Most of the responders (n=555; 89%) also added their own barriers as other option. Among the additional barriers, consultation time constraint (n=24; 28%) and uncertain benefits of and interests in health apps (n=19; 21%) were the leading reasons as to why the use of apps in daily clinical practice would be a challenge, whereas cost (n=3; 3%) was not rated as a major barrier.

When asked what would help them to recommend health apps to patients more often, the top answers were more knowledge, awareness, and training on health apps (n=243; 30%), a list of safe and effective apps provided or endorsed by reliable authority such as the RACGP (n=224; 28%), and quality, benefits, and evidence of apps (n=92; 12%). Time, integration into electronic medical software, access to better internet and mobile services, patients’ motivation, handouts, and practice incentives were all rated as of the least importance (less than 25 each, 1%-5%; Table 2).

The preferred ways to receive training on effective apps and app evaluation were permanent online video training material or webinars that the doctors could watch on their own time and pace (n=303; 33%). Other choices included podcasts (n=137; 15%), animation (n=101; 11%), face-to-face training (n=114; 12%), and other reading materials such as newsletters and articles (n=90; 10%).

**Table 2:** Facilitators of app prescription as responses to question “What would help you to recommend health apps to patients more often?” A total of 683 replies were coded into 800 answers. All other themes (n=9) had less than 25 answers each to support them.

Theme	Example comments
More awareness or knowledge or training (n=243)	“Educate us before we recommend to patients.”; “Myself getting more familiar about it and for me to learn what it is all about.”
List of approved or vetted apps (n=224)	“A clear directive, guideline about which are validated safe and useful”; “recommendation from respected advisors, for example, the Royal Australian College of GPs, National Prescribing Service”
Evidence or benefits or quality (n=92)	“Evidence for its use—case in the field.”
Nothing (n=44)	“Nothing. I recommend to my patients that they get away from screens and go and do some exercise, appreciate nature, and breathe some fresh air and RACGP should do the same instead of apparently trying to encourage everyone, doctors, and patients to increase their screen time.”
More time (n=39)	“More time during a consult to discuss benefits...however it starts to make us a ‘Telstra^a^ shop’ and not a GP practice.”
Practice incentive (n=29)	“Incentives—costs involved in recommending the apps—the ones I do recommend I research myself and spent time and money to do so.”
Integration with practice software (n=26)	“If the health information software used could pick up on coded diagnoses in patient's clinical information system and recommend a trustworthy app for the relevant medical conditions, it would be most beneficial.”

^a^Telstra is an Australian telecommunications company.

## Discussion

This study provides insights into the current adoption of smartphone health apps by Australian GPs. We found that two-thirds of the GPs use apps professionally, and at least half are recommending apps to patients. Mindfulness and mental health apps were most commonly suggested. Majority of the GPs were not aware of, and thus not using, evidence-based health apps that are included in the free RACGP resource HANDI. The biggest barriers to app prescription were the lack of knowledge of effective apps and the lack of trustworthy source to access them. To overcome these barriers, GPs expressed their need for a list of safe and effective apps from a trustworthy source and more training on health apps in the form of permanent online video training material or webinar that they could watch in their own time and pace.

The 2017 RACGP Technology Survey results were similar to the preceding year’s in terms of smartphone health app usage, most commonly used and recommended apps, and barriers to technology adoption, thus validating the trends [13]. Studies from several other countries reported that anywhere between 20% to 75% of health professionals use mHealth apps for their patients [8-11], which is comparable with the 50% use by Australian GPs. The main barriers we identified were also reflective of the barriers health professionals face around the world. The surveys indicate that health professionals recognize the potential benefit of smartphone health apps for self-management of health conditions and would like to use them in their work. However, they lack the knowledge, time, and skills to evaluate, find and recommend evidence-based apps, and therefore, they need help and guidance from the professional organizations and policy makers to overcome these barriers.

It is important that solutions to the barriers are unique to each country’s health care structures and health care professionals’ demands. For example, New Zealand’s Health Navigator website hosts a health app library set up by a GP organization and health care providers who use the apps can curate and provide feedback [5]. United Kingdom’s NHS not only provides Web-based apps library for doctors, but also introduced an app prescription platform for the GPs [17]. Similarly, for Australia, there is an opportunity for a professional organization such as the RACGP to lead the way to address the major barriers identified in this study. Although the inclusion of mHealth apps in the HANDI project is a starting place, more work needs to be done to raise awareness and profile of this initiative. Furthermore, integration of approved apps into the electronic medical systems to streamline the usability, as well as provide continuing professional development trainings for up-to-date information on mHealth apps, would enhance the use of evidence-based apps in clinical practice.

The strengths of this study include expanding on and improving previous year’s mHealth questions with more specific questions regarding evidence-based app adoption and barriers in general practice based on our qualitative research on GPs to obtain more comprehensive data on a nationally representative sample. Our response rate of 4.6% was similar to that of other mHealth app surveys undertaken on health professionals [9,10]. The completion rate of the mHealth section was uniformly high, although skipping questions was allowed.

The limitations of our survey study are the selection bias inherent in survey studies and the low response rate. However, the median age, median years practiced, and geographical distribution data of the GPs in our study were comparable with those of the national GP workforce data [16], thus supporting the demographic representativeness of our study population. A reason for the low response rate could be survey fatigue because of the fact that the mHealth questions analyzed here were a part of a larger survey on technological innovation in general practice [18]. The challenge for conducting survey studies on medical professionals is a balancing act between conducting a dedicated survey only focusing on single topics and the burdening of GPs with yet another survey. To increase the response rate of surveys that involve medical professionals, certain strategies could be undertaken such as offering more attractive incentives to participate and randomly sampling the cohort to send surveys and other study offers sparingly.

mHealth apps have a unique niche in the future of health care. However, the evidence of their effectiveness, safety, and usability issues are challenged by both the fast-evolving nature of the software and commercial aspects of the technology that can be easily exploited. Health care professionals need guidance on the quality of mHealth apps to assist in their adoption into clinical practice. In the absence of notable initiatives from government or private sectors to regulate app quality and safety, professional organizations must take the lead to address this challenge.

## Abbreviations

GP: general practitioner
HANDI: Handbook of Non-Drug Interventions
mHealth: mobile health
RACGP: Royal Australian College of General Practitioners
